# Supplementary material for: Evidence for increased parallel information transmission in human brain networks compared to macaques and male mice
Source: Nat Commun. 2023 Dec 11;14:8216. doi: 10.1038/s41467-023-43971-z (PMC10713651; doi:10.1038/s41467-023-43971-z)
Supplement: Supplementary file 3 — Reporting Summary [file 41467_2023_43971_MOESM3_ESM.pdf]

nature portfolio

Corresponding author(s): Alessandra GRIFFA, Enrico AMICO

Last updated by author(s): Oct 25, 2023

Reporting Summary

Nature Portfolio wishes to improve the reproducibility of the work that we publish. This form provides structure for consistency and transparency in reporting. For further information on Nature Portfolio policies, see our Editorial Policies and the Editorial Policy Checklist.

Statistics

For all statistical analyses, confirm that the following items are present in the figure legend, table legend, main text, or Methods section.

n/a	Confirmed	
☐	☒	The exact sample size (*n*) for each experimental group/condition, given as a discrete number and unit of measurement
☐	☒	A statement on whether measurements were taken from distinct samples or whether the same sample was measured repeatedly
☐	☒	The statistical test(s) used AND whether they are one- or two-sided<br>*Only common tests should be described solely by name; describe more complex techniques in the Methods section.*
☐	☒	A description of all covariates tested
☐	☒	A description of any assumptions or corrections, such as tests of normality and adjustment for multiple comparisons
☐	☒	A full description of the statistical parameters including central tendency (e.g. means) or other basic estimates (e.g. regression coefficient) AND variation (e.g. standard deviation) or associated estimates of uncertainty (e.g. confidence intervals)
☐	☒	For null hypothesis testing, the test statistic (e.g. *F*, *t*, *r*) with confidence intervals, effect sizes, degrees of freedom and *P* value noted<br>*Give P values as exact values whenever suitable.*
☒	☐	For Bayesian analysis, information on the choice of priors and Markov chain Monte Carlo settings
☐	☒	For hierarchical and complex designs, identification of the appropriate level for tests and full reporting of outcomes
☐	☒	Estimates of effect sizes (e.g. Cohen's *d*, Pearson's *r*), indicating how they were calculated

Our web collection on statistics for biologists contains articles on many of the points above.

Software and code

Policy information about availability of computer code

Data collection	No software was used to acquire the data.
Data analysis	Custom code was implemented in Matlab R2019b.<br>The code to reproduce the main results and figures is available through a public GitHub repository (https://github.com/agriffa/BrainComm_mammalian_evolution), as detailed in the Code Availability statement.

For manuscripts utilizing custom algorithms or software that are central to the research but not yet described in published literature, software must be made available to editors and reviewers. We strongly encourage code deposition in a community repository (e.g. GitHub). See the Nature Portfolio guidelines for submitting code & software for further information.

Data

Policy information about availability of data

All manuscripts must include a data availability statement. This statement should provide the following information, where applicable:

- Accession codes, unique identifiers, or web links for publicly available datasets
- A description of any restrictions on data availability
- For clinical datasets or third party data, please ensure that the statement adheres to our policy

All data used in this study are available through open-source repositories. The human h-HCP dataset is available at https://db.humanconnectome.org. The macaque q-NCS dataset is available through the Primate Data Exchange (PRIME-DE) initiative at https://fcon_1000.projects.nitrc.org/indi/indiPRIME.html. The macaque q-TVB

dataset is available at OpenNEURO (https://openneuro.org/datasets/ds001875/versions/1.0.3). The mouse m-GG dataset is available at https://data.mendeley.com/datasets/np2fx99hn6/2. The mouse m-AD3 dataset is available at OpenNEURO (https://openneuro.org/datasets/ds001890/versions/1.0.1). The mouse m-CSD1 dataset is available at the XNAT Data Repository https://central.xnat.org/ (Project_ID: CSD_MRI_MOUSE). A sample dataset generated in this study from open-source raw and processed data, including brain k-shortest paths and mutual information matrices of the three species, is available at the GitHub public repository https://github.com/agriffa/BrainComm_mammalian_evolution. Source data of all figures are provided as Source Data files. Source Data are provided with this paper.

Research involving human participants, their data, or biological material

Policy information about studies with human participants or human data. See also policy information about sex, gender (identity/presentation), and sexual orientation and race, ethnicity and racism.

Reporting on sex and gender	This study includes 100 biologically unrelated healthy human subjects, of which 36 females and 64 males. Sex was determined based on self-reporting within the Human Connectome Project framework. Sex was not considered in study design. This study investigates high-level brain network organization principles across different mammalian species. There is no specific hypothesis on sex-specific features at this level of investigation.
Reporting on race, ethnicity, or other socially relevant groupings	No race, ethnicity or other socially relevant grouping was investigated in this study.
Population characteristics	Human participants included in this study are 100 biologically unrelated healthy adults, 36 females and 64 males, mean age 29.1 +/- 3.7 years, recruited in the framework of the Human Connectome Project (HCP).
Recruitment	Subjects were selected from the HCP database with the following criteria: age between 22 and 36 years, unrelated subjects, available structural MRI data, functional MRI data (resting-state), and diffusion-weighted MRI data.
Ethics oversight	All experiments were reviewed and approved by the local institutional ethical committee (Swiss Ethics Committee on research involving humans). Informed consent forms, including consent to share de-identified data, were collected for all subjects (within the HCP) and approved by the Washington University Institutional Review Board. All methods were carried out in accordance with relevant guidelines and regulations.

Note that full information on the approval of the study protocol must also be provided in the manuscript.

Field-specific reporting

Please select the one below that is the best fit for your research. If you are not sure, read the appropriate sections before making your selection.

[x] Life sciences [ ] Behavioural & social sciences [ ] Ecological, evolutionary & environmental sciences

For a reference copy of the document with all sections, see nature.com/documents/nr-reporting-summary-flat.pdf

Life sciences study design

All studies must disclose on these points even when the disclosure is negative.

Sample size	100 human, 9 macaque, and 10 mouse experimental subjects were included in the main analysis. Moreover, three replication datasets were included for a total of 100 human, 9 macaque, and 71 mouse experimental subjects. The sample size was selected according to open-source data availability, and based on typical sample size in state-of-the-art cross-species studies. Given the explorative nature of this study, it was not possible to hypothesize a priori an effect size and determine a priori a sample size.
Data exclusions	No data were excluded from the analyses.
Replication	Three replication datasets were included to test results' reproducibility and assess the impact of dataset-specific factors such as anesthesia and magnetic resonance imaging (MRI) acquisition parameters. Moreover, main results were replicated including different numbers of experimental subjects.
Randomization	This study investigate high-level brain network organizational principle at group-level. For this reason, there was no allocation of subjects to distinct experimental groups.
Blinding	There was no group allocation of experimental subjects.

Reporting for specific materials, systems and methods

We require information from authors about some types of materials, experimental systems and methods used in many studies. Here, indicate whether each material, system or method listed is relevant to your study. If you are not sure if a list item applies to your research, read the appropriate section before selecting a response.

Materials & experimental systems

n/a	Involved in the study
x	Antibodies
x	Eukaryotic cell lines
x	Palaeontology and archaeology
	x Animals and other organisms
x	Clinical data
x	Dual use research of concern
x	Plants

Methods

n/a	Involved in the study
x	ChIP-seq
x	Flow cytometry
	x MRI-based neuroimaging

Animals and other research organisms

Policy information about studies involving animals; ARRIVE guidelines recommended for reporting animal research, and Sex and Gender in Research

Laboratory animals	- q-NCS macaque dataset: 9 adult rhesus macaque monkeys (Macaca mulatta; 2 females) aged between 5 and 14 years<br>- q-TVB macaque dataset: 9 adult male rhesus macaque monkeys (8 Macaca mulatta, 1 Macaca fascicularis) aged between 4 and 8 years<br>- m-GG mouse dataset: 10 C57Bl6/J adult male mice, < 6 months old<br>- m-AD3 mouse dataset: 10 male wild-type mice aged 6 months<br>- m-CSD1 dataset: 51 male wild-type mice aged 3 months
Wild animals	No wild animals' data were used.
Reporting on sex	Sex was not considered in study design. This study investigates high-level brain network organization principles across different mammalian species. There is no specific hypothesis on sex-specific features at this level of investigation. All mouse experimental subjects were males.
Field-collected samples	No field-collected samples were used.
Ethics oversight	All data used in this work belong to publicly available databases. All methods were carried out in accordance with relevant guidelines and ethical regulations and have been previously described (detailed references are provided in the manuscript).

Note that full information on the approval of the study protocol must also be provided in the manuscript.

Plants

Seed stocks	No plants involved in this study.
Novel plant genotypes	No plants involved in this study.
Authentication	No plants involved in this study.

Magnetic resonance imaging

Experimental design

Design type	Resting-state.
Design specifications	- h-HCP human dataset: resting-state fMRI data were acquired in one run of approximately 15 minutes, with eye open with relaxed fixation on a projected bright cross-hair on a dark background. Within the recording session, oblique axial acquisitions alternated between phase encoding in a right-to-left (RL) direction and phase encoding in a left-to-right (LR) direction, for a total of 2 acquisitions per subject.<br><br>- q-NCS macaque dataset: resting-state data were acquired in two runs of approximately 11 minutes each. The two runs were concatenated. Animals were scanned awake.<br><br>- q-TVB macaque dataset: fMRI data were acquired in one run of approximately 10 minutes. During the recording, animals were lightly anesthetized before their scanning session and anesthesia was maintained using 1-1.5% isoflurane.

- m-GG mouse dataset: resting-state data were acquired in one run of approximately 32 minutes. Animal were subject to surgery for headposts placement, MRI habituation and awake fMRI acquisition.

- m-AD3 mouse dataset: fMRI data were acquired in one run of approximately 10 minutes. Animals were anesthetized with 4% isoflurane before their scanning session and maintained with 0.5% isoflurane and a 0.05 mg/kg/h medetomidine infusion.

- m-CSD1 mouse dataset: fMRi data were acquired in one run of approximately 6 minutes. Animals were anesthetized with 3.5% isoflurane before their scanning session and maintained with 0.5% isoflurane and a 0.05 mg/kg/h medetomidine infusion.

We refer to the different datasets' documentation for complete details on experimental design.

Behavioral performance measures

No behavioral performance measures were used.

Acquisition

Imaging type(s)

Functional, structural, and diffusion-weighted MRI.

Field strength

- h-HCP human dataset: 3T Siemens Prisma scanner
- q-NCS macaque dataset: 4.7T vertical Bruker primate-dedicated scanner
- q-TVB macaque dataset: 7T Siemens MAGNETOM head scanner
- m-GG mouse dataset: 7T Bruker BioSpin scanner
- m-AD3 mouse dataset: 11.75T Bruker BioSpin scanner
- m-CSD1 mouse dataset: 9.4T Bruker BioSpin scanner

Sequence & imaging parameters

- h-HCP human dataset: MRI scans were performed on a 3T Siemens Prisma scanner and included the following sequences: Structural MRI: 3D Magnetization Prepared Rapid Acquisition with Gradient Echoes (MPRAGE) T1-weighted, TR = 2400 ms, TE = 2.14 ms, TI = 1000 ms, flip angle = 8°, FOV = 224 × 224, voxel size = 0.7 mm isotropic. One session of 15 min resting-state functional MRI (fMRI): gradient-echo EPI, TR = 720 ms, TE = 33.1 ms, flip angle = 52°, FOV = 208 × 180, voxel size = 2 mm isotropic, recorded with two phase-encoding directions (right-left and left-right).

- q-NCS macaque dataset: Structural MRI: Modified Driven Equilibrium Fourier Transform (MDEFT) T1-weighted, TR = 2000 ms, TE = 3.75 ms, TI = 750 ms, voxel size = 0.6 x 0.6 x 0.62 mm3. Two runs of 6.5 min resting-state fMRI: TR = 2600 ms, TE = 17 ms, voxel size = 1.2 mm isotropic.

- q-TVB macaque dataset: Structural MRI: 3D MPRAGE T1-weighted sequence, 128 slices, voxel size = 0.5 mm isotropic. Diffusion-weighted MRI: EPI sequence, 24 slices, b-value = 1000 s/mm2, 64 directions, recorded with two opposite phase-encoding directions. One session of 10 min resting-state functional MRI (fMRI): 2D multiband EPI sequence, TR = 1000 ms, 42 slices, 1 X 1 X 1.1 mm3 voxel size.

- m-GG mouse dataset: 32-min resting-state fMRI recording: single-shot EPI sequence, TR = 1000 ms, TE = 15 ms, flip angle = 60°, voxels size = 0.23 x 0.23 x 0.6 mm3.

- m-AD3 mouse dataset: Structural MRI: spin-echo turboRARE sequence, TR = 2750 ms, TE = 30 ms, FOV = 17 x 11 mm2, matrix dimension = 200 x 100 voxels, slice thickness = 0.35 mm. One session of 10 min resting-state functional MRI (fMRI): gradient-echo EPI sequence, TR = 1000 ms, TE = 15 ms, matrix dimension = 90 x 60 voxels.

- m-CSD1 mouse dataset: 6-min resting-state fMRI recording: gradient-echo EPI sequence, TR = 1000 ms, TE = 9.2 ms, flip angle = 90°, field of view = 20 x 17.5 mm2, matrix size = 90 x 70 voxels, slice thickness = 0.5 mm.

Area of acquisition

Whole brain

Diffusion MRI ☒ Used ☐ Not used

Parameters

h-HCP human dataset only: diffusion-weighted MRI: spin-echo Echo-Planar Imaging (EPI), TR = 5520 ms, TE = 89.5 ms, flip angle = 78°, FOV = 208 × 180, 3 shells of b-value = 1000, 2000, 3000 s/mm2 with 90 directions plus 6 b-value = 0 s/mm2 acquisitions.

Preprocessing

Preprocessing software

Matlab R2019b.

Normalization

Nonlinear spatial normalization to species-specific templates was performed to bring species-specific cortical atlases to individual native space.

Normalization template

- Human dataset: Montreal Neurological Institute (MNI) standard space
- Macaque datasets: F99 macaque template
- Mouse datasets: Allen brain atlas mouse template

Noise and artifact removal

T1-weighted volumes were denoised, skull-stripped, corrected for bias field artefacts, spatially normalized to species-specific template (nonlinear warping), and linearly registered to native space. fMRI volumes were coregistered, corrected for

	nuissance signals including motion signals, average white matter and cerebrospinal fluid signals, and band-pass filtered to the band 0.01-0.15 Hz.
Volume censoring	No volume censoring was applied.

Statistical modeling & inference

Model type and settings	Group-comparisons were performed using two-sided Mann-Whitney U test, two sample Kolmogorov-Smirnov test, and Kruskal-Wallis test. In addition, four null models were specifically designed for our analyses: the first one involves the randomly shuffling of the raw fMRI time series across brain regions while preserving the original structural connectivity information; the second one was defined by populating the network nodes with iid Gaussian noise with mean 0, variance 1, and the same number of time points as in experimental data; the third one was developed for mutual information (MI)-based functional connectomes, which preserves their spatial autocorrelation with respect to the underlying distance-weighted structural connectivity matrix; a forth one involves the shuffling of subjects' labels for brain fingerprinting analysis.
Effect(s) tested	Difference between medians of two distributions; difference between two datasets' distributions; effect of species on variables of interest; significance of results and of brain spatial patterns against specifically designed null models which preserve certain data statistics.

Specify type of analysis: [x] Whole brain [ ] ROI-based [ ] Both

Statistic type for inference<br>(See Eklund et al. 2016)	None, not relevant.
Correction	None, not relevant.

Models & analysis

n/a	Involved in the study
[ ]	[x] Functional and/or effective connectivity
[ ]	[x] Graph analysis
[x]	[ ] Multivariate modeling or predictive analysis

Functional and/or effective connectivity	Z-scored time series were used to compute mutual information values between brain region pairs.
Graph analysis	We introduced a graph weighting (the parallel communication scores) which jointly considers the brain structural connectivity architecture (weighted by the Euclidean distance between region pairs), the structural short paths layout (k-shortest paths), and the sequences of fMRI-derived mutual information values along the structural paths.
